# Supplementary figures and images for: The Causal Relationship Between Choline Metabolites and Acute Acalculous Cholecystitis: Identifying ABCG8 as Colocalized Gene
Source: Nutrients. 2024 Oct 22;16(21):3588. doi: 10.3390/nu16213588 (PMC11547454; doi:10.3390/nu16213588)

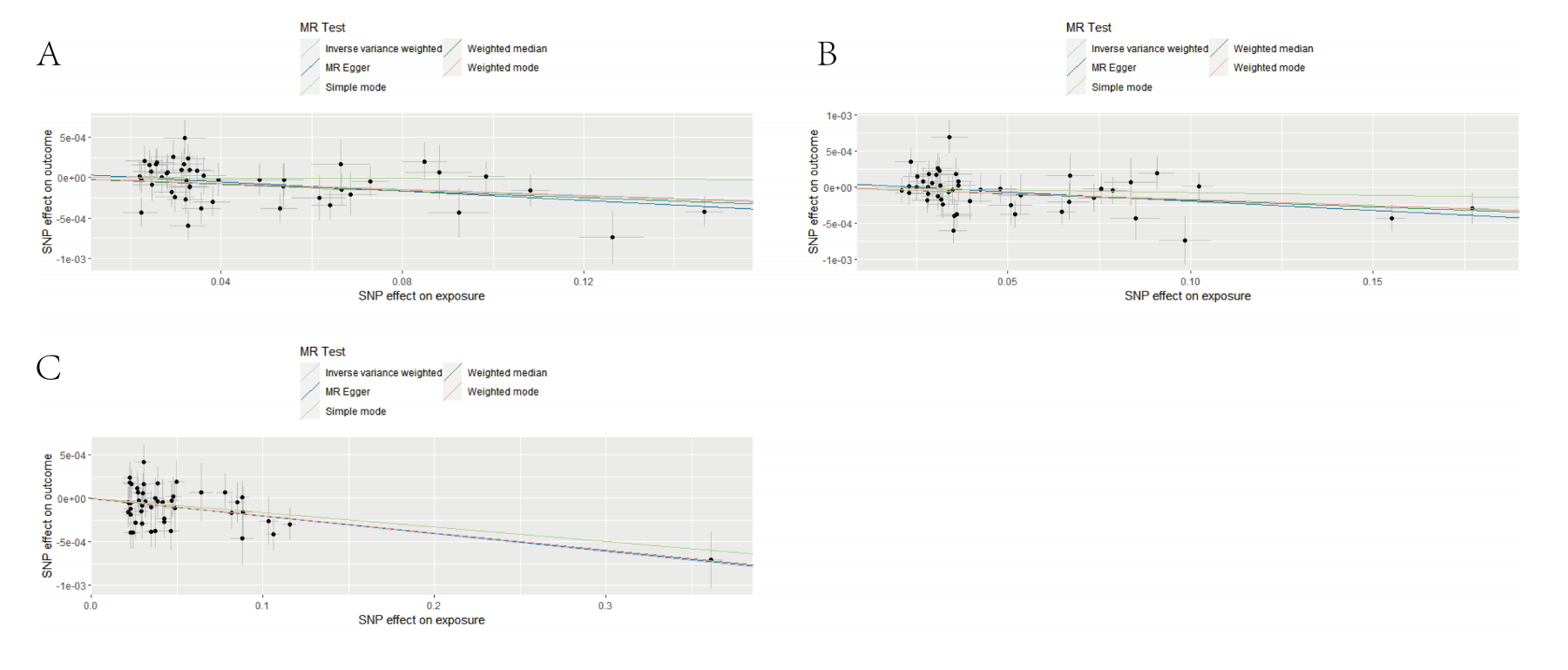

Supplement: Supplementary file 1 [file nutrients-16-03588-s001.zip › Supplementary Materials/Supplementary Figure S1.tif]

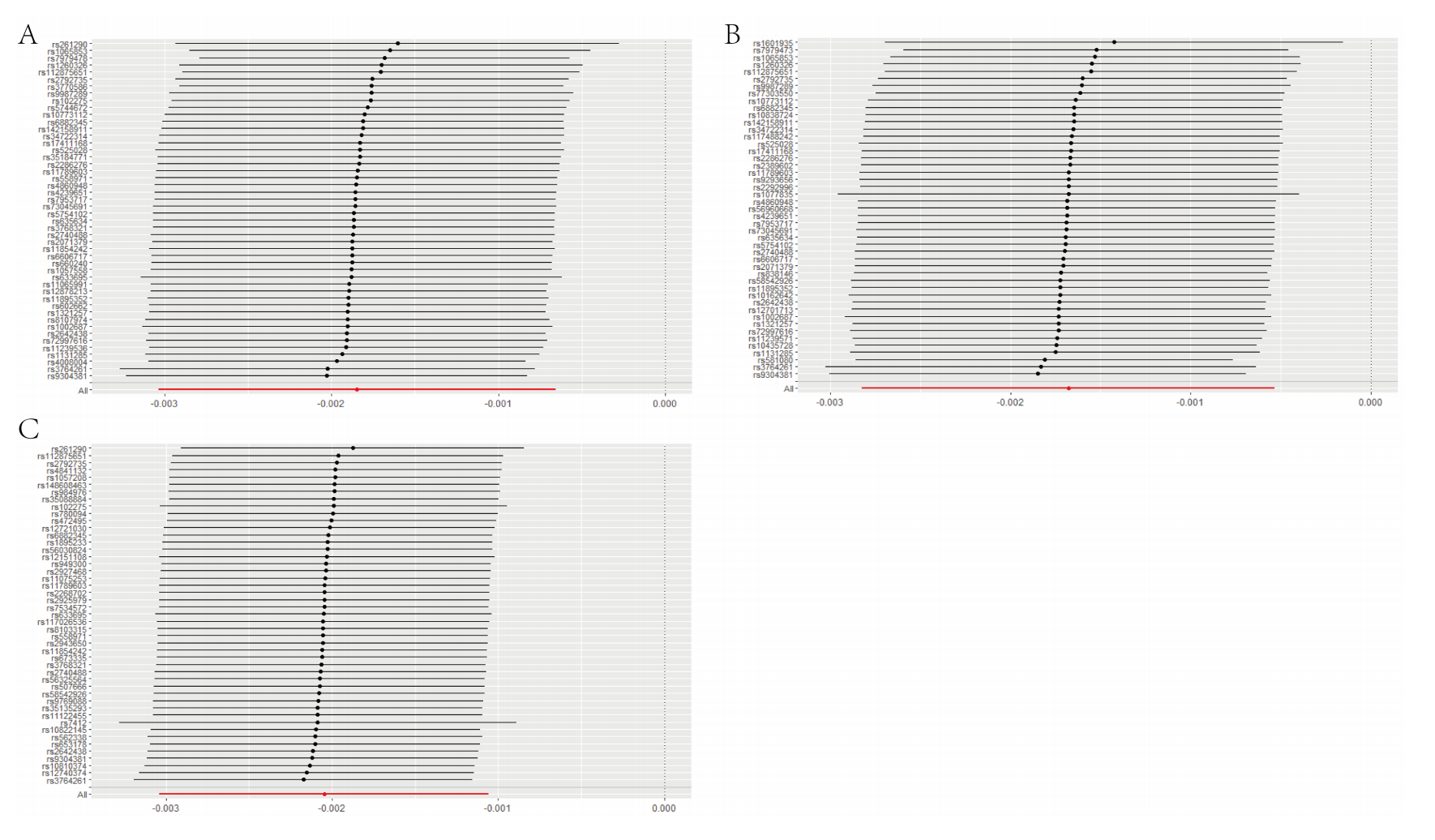

Supplement: Supplementary file 1 [file nutrients-16-03588-s001.zip › Supplementary Materials/Supplementary Figure S2.tif]

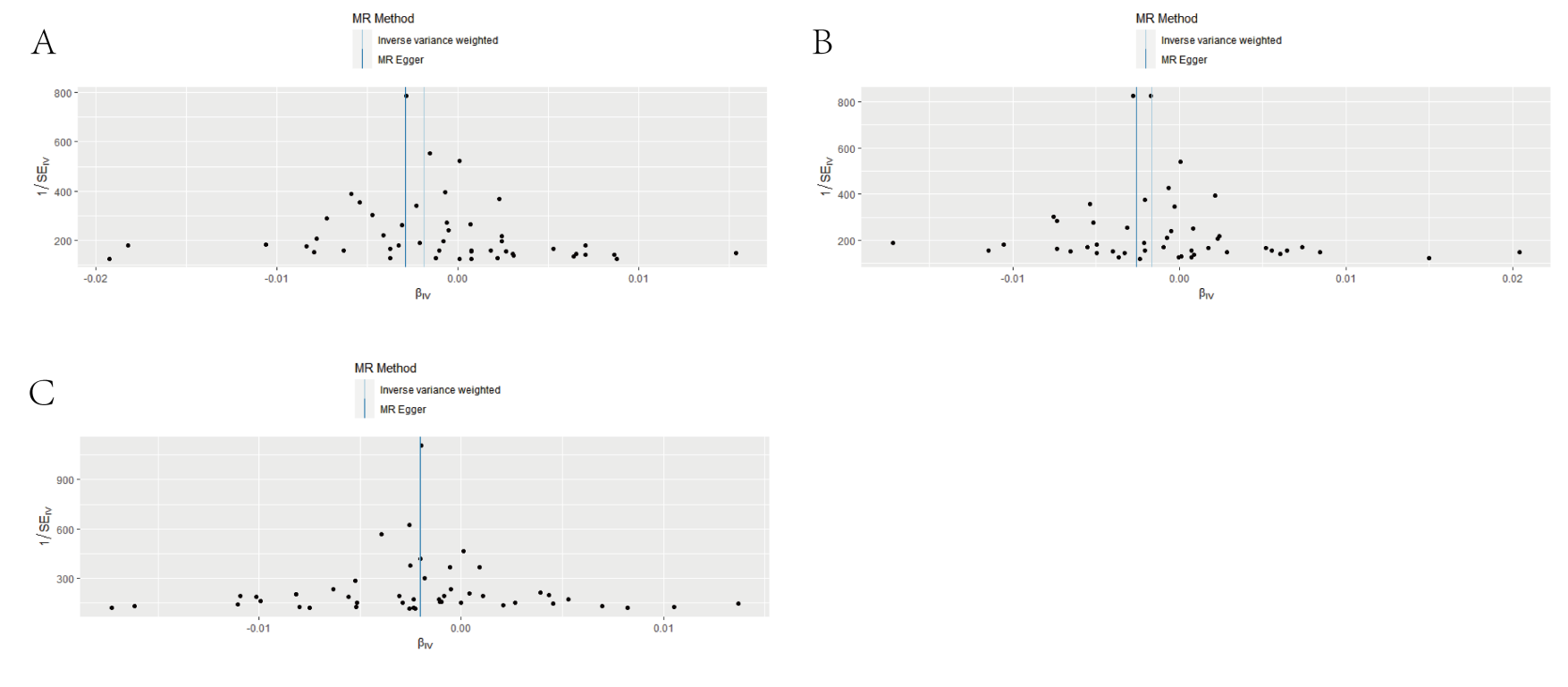

Supplement: Supplementary file 1 [file nutrients-16-03588-s001.zip › Supplementary Materials/Supplementary Figure S3.tif]
